# Nucleated red blood cells as a prognostic marker for mortality in patients with SARS-CoV-2-induced ARDS: an observational study

**DOI:** 10.1186/s44158-024-00174-2

**Published:** 2024-06-28

**Authors:** Anna Kirsch, Felix Niebhagen, Miriam Goldammer, Sandra Waske, Lars Heubner, Paul Petrick, Andreas Güldner, Thea Koch, Peter Spieth, Mario Menk

**Affiliations:** 1 Department of Anesthesiology and Intensive Care Medicine, Faculty of Medicine, University Hospital Carl Gustav Carus and Carl Gustav Carus, TU Dresden, Dresden, Germany; 2 Institute for Medical Informatics and Biometry, Carl Gustav Carus Faculty of Medicine, TU Dresden, Dresden, Germany

**Keywords:** NRBC, ARDS, COVID-19, SARS-CoV-2, Erythroblasts, Mortality, ICU, Prognosis, Prognostic marker

## Abstract

**Background:**

The presence of nucleated red blood cells (NRBCs) in the peripheral blood of critically ill patients is associated with poor outcome. Evidence regarding the predictive value of NRBCs in patients with SARS-CoV-2-induced acute respiratory distress syndrome (ARDS) remains elusive. The aim of this study was to evaluate the predictive validity of NRBCs in these patients.

**Methods:**

Daily NRBC values of adult patients with SARS-CoV-2-induced ARDS were assessed and their predictive validity for mortality was statistically evaluated.

A cut-off level based on the patient’s maximum NRBC value during ICU stay was calculated and further specified according to Youden’s method. Based on this cut-off value, further analyses such as logistic regression models and survival were performed.

**Results:**

413 critically ill patients with SARS-CoV-2-induced ARDS were analyzed. Patients who did not survive had significantly higher NRBC values during their ICU stay compared to patients who survived (1090/µl [310; 3883] vs. 140/µl [20; 500]; *p* < 0.0001). Patients with severe ARDS (*n* = 374) had significantly higher NRBC values during ICU stay compared to patients with moderate ARDS (*n* = 38) (490/µl [120; 1890] vs. 30/µl [10; 476]; *p* < 0.0001).

A cut-off level of NRBC ≥ 500/µl was found to best stratify risk and was associated with a longer duration of ICU stay (12 [8; 18] vs. 18 [13; 27] days; *p* < 0.0001) and longer duration of mechanical ventilation (10 [6; 16] vs. 17 [12; 26] days; *p* < 0.0001). Logistic regression analysis with multivariate adjustment showed NRBCs ≥ 500/µl to be an independent risk factor of mortality (odds ratio (OR) 4.72; 95% confidence interval (CI) 2.95–7.62, *p* < 0.0001). Patients with NRBC values below the threshold of 500/µl had a significant survival advantage over those above the threshold (median survival 32 [95% CI 8.7–43.3] vs. 21 days [95% CI 18.2–23.8], log-rank test, *p* < 0.05).

Patients who once reached the NRBC threshold of ≥ 500/µl during their ICU stay had a significantly increased long-term mortality (median survival 489 days, log-rank test, *p* = 0.0029, hazard ratio (HR) 3.2, 95% CI 1.2–8.5).

**Conclusions:**

NRBCs predict mortality in critically ill patients with SARS-CoV-2-induced ARDS with high prognostic power. Further studies are required to confirm the clinical impact of NRBCs to eventually enhance decision making.

**Supplementary Information:**

The online version contains supplementary material available at 10.1186/s44158-024-00174-2.

## Background

Extensive research has focused on understanding SARS-CoV-2 (severe acute respiratory syndrome coronavirus type), including its progression, therapeutic targets and potential prognostic factors [1]. Approximately 10–15% of patients with SARS-CoV-2 require hospitalization, of which 20–30% develop life-threatening manifestations such as acute respiratory distress syndrome (ARDS) necessitating intensive care unit (ICU) treatment [1–3]. Despite recent advances, ICU mortality in these patients remains high, varying from 20% to more than 50% depending on various factors such as geographical location, demographic characteristics, and underlying health conditions [4, 7]. Advanced age, obesity and hypertension have been identified as independent risk factors for severe SARS-CoV-2, with mechanical ventilation correlating with particularly high mortality rates [4–8]. Furthermore, elevated inflammatory markers such as procalcitonin (PCT), C-reactive protein (CRP) and white blood cell count have been found to be associated with disease severity and mortality [5, 6]. These markers may serve as predictive parameters to some extent. However, the identification of reliable prognostic biomarkers seems crucial for optimizing patient management and resource allocation, especially in critically ill patients with SARS-CoV-2-induced ARDS, but remains challenging [9–12].

Nucleated red blood cells (NRBCs), precursors of red blood cells, are typically confined to the bone marrow in healthy adults and older children [13]. Their presence in the peripheral blood indicates a disruption of the blood-bone marrow barrier due to hypoxemia or excessive cytokine release, and has been observed in various pathological conditions, including cancer, congestive heart failure and inflammation [16–18]. Importantly, the presence of NRBCs in critically ill patients has been consistently associated with increased mortality rates and poorer clinical outcomes [14–16].

Despite this knowledge, evidence regarding the predictive value of NRBCs in SARS-CoV-2-induced ARDS remains limited. Therefore, the primary objective of this study was to assess the prognostic utility of NRBCs in these patients. In addition, we aimed to investigate whether sustained elevation of NRBC levels above a critical plasma concentration threshold influences mortality. Furthermore, we seek to establish clinically relevant thresholds of NRBC levels as early predictors of mortality and clinical outcomes.

## Methods

### Setting and patients

This retrospective study was conducted at the ICU of the Department of Anesthesiology and Intensive Care Medicine, University Hospital Carl Gustav Carus Dresden, Germany; a national referral center for the treatment of ARDS. All adult patients aged ≥ 18 years with SARS-CoV-2-induced ARDS admitted to the ICU between March 2020 and March 2022 were enrolled. Patients were screened for SARS-CoV-2 infection and treated according to local hospital standard operating procedures based on the recommendations of the German COVID-19 guideline and the Robert Koch Institute guidelines (COVRIIN, RKI) [17]. The study was approved by the local ethics committee (BO-EK-374072021).

### Data collection

Patient data were retrieved from the local electronic patient data management systems (ORBIS, Dedalus, Bonn, Germany and ICM, Dräger Medical, Lübeck, Germany). Demographic data such as sex, age, height, weight, and scores such as the Charlson Comorbidity Index (CCI) were recorded upon ICU admission [18–20]. ARDS was defined as mild, moderate or severe according to the Berlin definition [21]. Additional data included ICU mortality, ICU length of stay, the requirement for and of duration for extracorporeal membrane oxygenation (ECMO), parameters of mechanical ventilation such as mean airway pressure (Pmean), positive end-expiratory pressure (PEEP) and others, renal replacement therapy (RRT), and routine laboratory parameters such as C-reactive protein (CRP), procalcitonin (PCT), white blood cell count and bilirubin.

### Laboratory tests, NRBCs

NRBC values were assessed by daily routine laboratory EDTA blood samples throughout the ICU stay and are reported in (n)/µl. In case of multiple NRBC values per day, the maximum value was selected for analysis. The highest NRBC value for each patient was also used for analyses over time, including receiver operating characteristics (ROC) curves, linear regression, COX regression and survival analysis. Mean values of the assessed maximum NRBC for each day were calculated and are reported for the first 30 days of ICU stay. In addition, the increase or decrease in NRBC (∆NRBC) for each patient for each day compared to the previous day was calculated as ∆NRBC_day x_ = NRBC_day x_ – NRBC_day x-1_; representing the absolute change of NRBC per 24 h. The resulting highest ∆NRBC value was then used again for similar analyses as described above.

### Endpoints

The primary endpoint was to determine a cut-off value of NRBC that predicts a significant increase in ICU mortality among patients with SARS-CoV-2-induced ARDS. Secondary endpoints included ICU mortality, ICU length of stay, survival after ICU (follow-up survey three months to two years after ICU discharge), the requirement for and duration of ECMO, mechanical ventilation, and RRT in relation to the cutoff value. In addition, we analyzed the impact of a specific increase of NRBC within a 24-h period on patient outcomes.

### Statistical analysis

Discrete variables are presented as absolute numbers, counts, or percentages as indicated. Continuous variables are presented as medians with 25th and 75th percentiles. Statistical differences between groups for demographic patient characteristics were assessed using Fisher’s exact test for categorical variables and using the Mann–Whitney U test for continuous variables. Daily NRBC and ∆NRBC values were evaluated and their predictive validity for mortality was calculated using ROC. A cut-off level representing the highest sum of sensitivity and specificity based on each patient’s maximum NRBC value during ICU stay was further specified according to Youden’s method, as described previously [22]. We chose Youden’s method because of its ease of use. We used the parametric approach to calculate a cut-off value. In applying Youden’s method, the ROC was first generated by calculating the sensitivity and specificity over a range of maximum NRBC values. For each NRBC value, the Youden index was calculated by using the following formula: Youden = sensitivity + specificity-1. The cut-off value corresponding to the highest Youden index was identified as the optimal cut-off, reflecting the highest sum of sensitivity and specificity. This threshold was then utilized to categorize patients into two groups, one above and one below the respective threshold. Multiple logistic regression analysis with stepwise backward selection was employed to carry out multivariate testing for factors that influence mortality. Survival was depicted using Kaplan–Meier estimates, which were tested for differences between groups using the log-rank test. In order to test multivariate for factors that influence survival, COX regression was applied with stepwise backward selection, including variables that showed a statistically significant impact in the univariate analyses. Differences between groups in clinical parameters over time were assessed using a mixed-linear model. Statistical analyses were performed using IBM SPSS Statistics, version 29 (SPSS, Chicago, IL, USA) and GraphPad PRISM version 10.1.1 (323) (San Diego, CA, USA). A two-tailed *p*-value < 0.05 was considered statistically significant. All tests should be understood as constituting exploratory analysis, such that no adjustments for multiple testing have been made.

## Results

### Patient characteristics

In total, 413 critically ill patients with SARS-CoV-2-induced ARDS were included in this analysis. NRBCs were found in 97.6% of patients. Thus, only 2.4% of the patients never had NRBCs detectable. Patients’ characteristics are presented in Table 1.

**Table 1:** Patient characteristics grouped by NRBC cut-off value ≥ 500/µl

	all patients (*n* = 413)	NRBC < 500/µl (*n* = 218)	NRBC ≥ 500/µl (*n* = 195)	*p*-value
*Demographics*				
Age (years)	62 (56; 69)	64 (56; 70)	61 (55; 68)	n.s
Male sex, *n* (%)	295 (71.4)	160 (73.4)	135 (69.2)	n.s
Body mass index (kg/m2)	29.4 (26.2; 34.4)	30.3 (26.2; 34)	29.4 (26.2; 35)	n.s
Septic shock at admission, *n* (%)	92 (22.3)	38 (17.43)	54 (27.7)	0.0177
ARDS severity, *n* (%)				0.0021*
Mild	1 (0.24)	1 (0.46)	0 (0)	
Moderate	38 (9.2)	29 (13.3)	9 (4.6)	
Severe	374 (90.55)	188 (86.2)	186 (95.4)	
*ICU characteristics*				
Spontaneous breathing at admission, *n* (%)	112 (27.1)	70 (32.1)	42 (21.5)	0.019
Intubated at admission, *n* (%)	301 (72.88)	148 (67.89)	153 (78.46)	0.0001
Tracheotomy at admission, *n* (%)	29 (7.02)	15 (6.88)	14 (7.18)	n.s
Mechanical ventilation (days)	14 (8; 21)	10 (6; 16)	17 (12; 26)	< 0.0001
ECMO, *n* (%)	178 (43.1)	52 (23.85)	126 (64.6)	< 0.0001
ECMO duration (hours)	326.29 (196.93; 569.78)	215.204 (141.7; 407.4)	351.57 (239.5; 601.0)	0.0006
ICU length of stay (days)	15 (10; 23)	12 (8; 18)	18 (13; 27)	< 0.0001
RRT,* n* (%)	161 (38.98)	40 (18.35)	121 (62.05)	< 0.0001
RRT duration (hours)	154.65 (61.34; 280.4)	145.87 (82.0; 269.9)	165.33 (58.0; 280.66)	n.s
ICU mortality, *n* (%)	206 (49.8)	64 (29.36)	142 (72.8)	< 0.0001
*Ventilation parameters*				
pH	7.38 (7.32; 7.44)	7.38 (7.33; 7.44)	7.37 (7.31; 7.43)	n.s
PaCO_2_ (kPa)	6.51 (5.78; 7.6)	6.47 (5.81; 7.38)	6.68 (5.7; 8.07)	n.s
P/F ratio	60.0 (45.0; 75.0)	67.5 (52.5; 82.5)	52.5 (45.0; 60.0)	< 0.0001
Pmean (cm H_2_O)	21 (18; 23)	20 (17; 23)	21 (19; 23)	0.0023
PEEP (cm H_2_O)	14 (12; 15)	14 (12; 15)	15 (13; 15)	0.0060
SpO_2_ (%)	93 (90; 96)	93 (90; 96)	93 (90; 96)	n.s
*Laboratory parameters*				
D-dimers max (ng/ml)	4000 (3771; 5185)	4000 (3296; 7179)	4000 (3961; 4951)	0.017
Bilirubin µmol/l	41 (17; 110)	22.5 (13; 48)	95 (37; 219)	< 0.0001
CRP (mg/l)	284.1 (203.2; 364.7)	242.1 (166.5; 321.6)	334.1 (244.9; 410.6)	< 0.0001
PCT (ng/ml)	3.37 (0.98; 10.5)	1.74 (0.54; 5.87)	7.74 (2.51; 15.2)	< 0.0001
IL-6 maximum value, (pg/ml)	508 (179; 2533)	312 (116; 635)	1535 (399; 7209)	< 0.0001
Leukocytes maximum value (GPt/L)	21.14 (16.49; 28.06)	18.31 (13.93; 22.6)	25.88 (20.06; 32.3)	< 0.0001
*Excerpt from pre-existing conditions*				
Arterial hypertension, *n* (%)	272 (65.86)	146 (66.97)	126 (64.6)	n.s
Cardiovascular diseases, *n* (%)	194 (46.97)	93 (42.66)	101 (51.79)	n.s
Neurovascular/Stroke/ICB, *n* (%)	31 (7.51)	17 (7.8)	14 (7.2)	n.s
CHD, *n* (%)	50 (12.11)	28 (12.8)	22 (11.3)	n.s
COPD, *n* (%)	18 (4.36)	12 (5.5)	6 (3.1)	n.s
Other chronic lung diseases, *n* (%)	39 (9.44)	21 (9.6)	18 (9.2)	n.s
Nicotine abuse, *n* (%)	34 (8.23)	20 (9.2)	14 (7.2)	n.s
Diabetes, *n* (%)	141 (34.14)	78 (35.8)	63 (32.3)	n.s
Chronic kidney disease, *n* (%)	40 (9.69)	18 (8.3)	22 (11.3)	n.s
Chronic dialysis, *n* (%)	11 (2.66)	3 (1.4)	8 (4.1)	n.s
Charlson Comorbidity Index (CCI)	2 (1;4)	3 (1; 4)	2 (1;4)	n.s

Discrete variables are presented as absolute numbers, median or percentage and were analyzed with Fisher’s exact test. Continuous variables are presented as median and (25; 75) percentiles and were analyzed with the Mann–Whitney U test for independent groups

n.s. = not significant

*NRBC* nucleated red blood cells, *ARDS* acute respiratory distress syndrome, *ICU* intensive care unit, *ECMO* extracorporeal membrane oxygenation, *RRT* renal replacement therapy, *PaCO*_*2*_ arterial partial pressure of carbon dioxide*, PaO*_*2*_ arterial partial pressure of oxygen, *SpO*_*2*_ peripheral oxygen saturation, *CRP* C-reactive protein, *PCT* Procalcitonin, *IL-6* Interleukin-6, *ICH* intracranial hemorrhage, *COPD* chronic obstructive pulmonary disease, *CHD* coronary heart disease

^*^significance for ARDS severity moderate vs. severe, mild was excluded due to *n* = 1

### NRBC maximum values and 30-day development

**Fig. 1:**
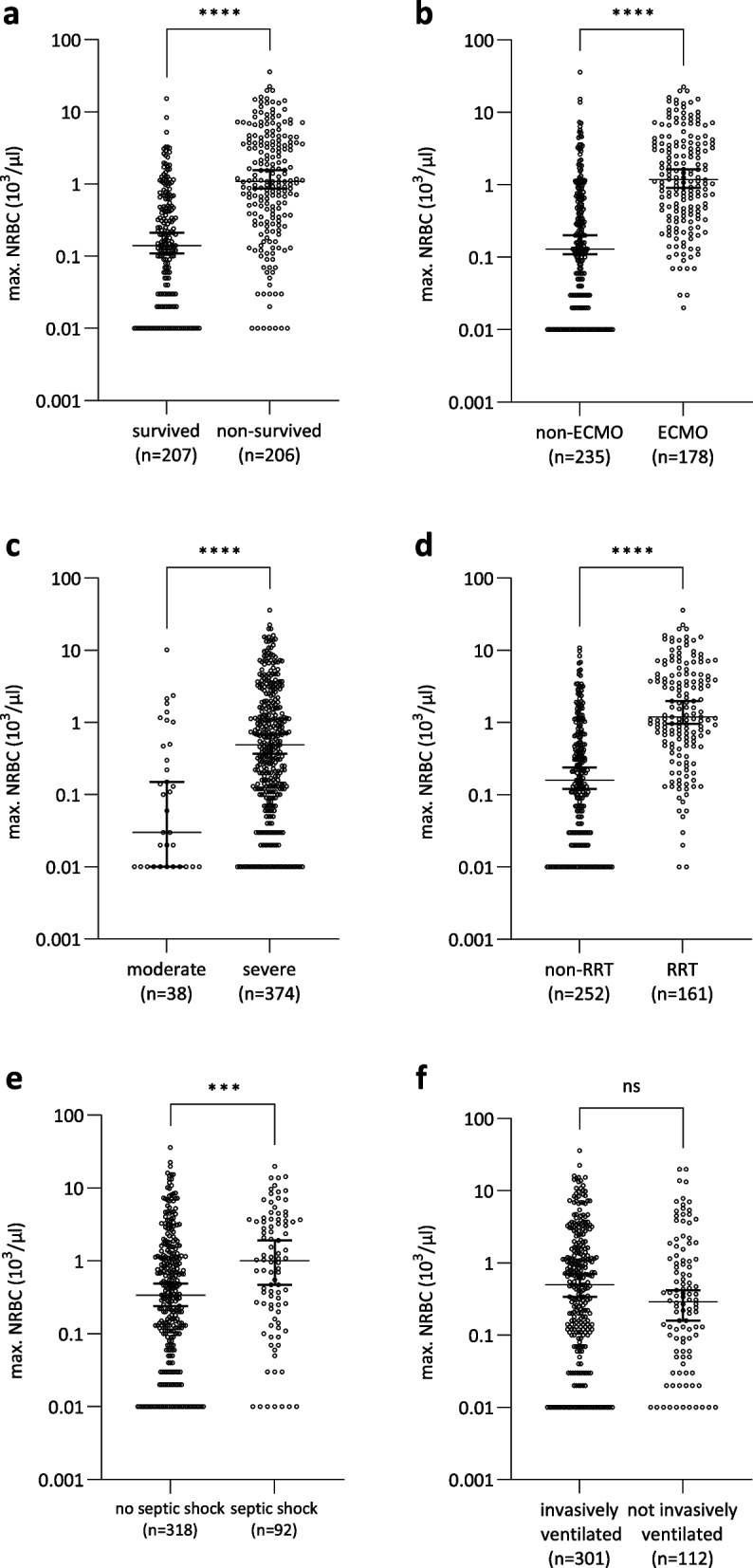
Maximum concentration of nucleated red blood cells (NRBCs) in patients with SARS-CoV-2-induced ARDS grouped by a survivors and non-survivors b implementation of ECMO and no ECMO c severity of lung failure according to the Berlin definition of ARDS (moderate, severe) (mild was excluded from further analysis due to n = 1) **d** implementation of renal replacement therapy (RRT) and no RRT **e** no septic shock and septic shock at admission **f** invasively ventilated and not invasively ventilated. N.s. as indicated, Mann–Whitney test, ***p < 0.001; ****p < 0.0001; *ns* not significant

Analysis of maximum NRBC values per day revealed notable differences between survivors (*n* = 207) and non-survivors (*n* = 206) during the ICU stay (median NRBC survivors: 140/µl [20; 500] vs. non-survivors: 1090/µl [310; 3883]; *p* < 0.0001) (Fig. 1a). Patients requiring ECMO exhibited significantly higher NRBC levels (median NRBC 1180/µl [413; 3755] vs. 130/µl [20; 690]; *p* < 0.0001), as did those requiring RRT (median NRBC 1190/µl [495; 4570] vs. 160/µl [30; 697]; *p* < 0.0001), compared to patients who did not require ECMO or RRT (Fig. 1b/d). In terms of ARDS severity according to the Berlin definition, patients with severe ARDS (*n* = 374) had significantly higher NRBC values during ICU stay compared to those with moderate ARDS (*n* = 38) (median 490/µl [120; 1890] vs. 30/µl [10; 476]; *p* < 0.0001) (Fig. 1c). There was only one patient with mild ARDS in this study population who was excluded from further analysis due to small sample size. Patients with septic shock (*n* = 92) at ICU admission demonstrated elevated NRBC levels compared to those without septic shock (*n* = 318) (median 950/µl [160; 3540] vs. 340/µl [70; 1208]; *p* < 0.001) (Fig. 1e). The difference in NRBC values at ICU admission between patients who were invasively ventilated (*n* = 301) and those who were not (*n* = 112) was not significant (median 500/µl [110; 1810] vs. 290/µl [53; 1145]; n.s.) (Fig. 1f).

**Fig. 2:**
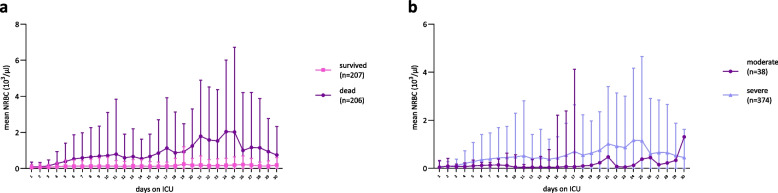
Development of mean NRBC in patients with SARS-CoV-2-induced ARDS during first 30 days on ICU grouped by **a** survivors and non-survivors and **b** severity of lung failure according to the Berlin definition of ARDS (moderate, severe) (mild was excluded from further analysis due to n = 1). Data are presented as mean ± standard deviation (SD); n = 413

Over the first 30 days in the ICU, mean NRBC values (of all peaks) per day were markedly lower in survivors compared to non-survivors, with significant differences observed over time (mean NRBC in survivors: median 136.8/µl [127.6; 176.2] vs. mean NRBC in non-survivors: median 775.8/µl [576.7; 1155];

*p* < 0.0001) (Fig. 2a). Patients with severe ARDS consistently exhibited higher mean NRBC values compared to those with moderate ARDS (severe ARDS median NRBC 501/µl [387.3; 669.4] vs. moderate ARDS median NRBC 100/µl [52.3; 167.5]; *p* < 0.0001) (Fig. 2b).

In our findings, comorbidities, such as cardiovascular disease, arterial hypertension, neurovascular disease, diabetes, chronic kidney disease, other pre-existing conditions and the CCI did not have a relevant impact on increased NRBC levels and COVID-19 outcomes.

#### NRBCs and mortality

**Fig. 3:**
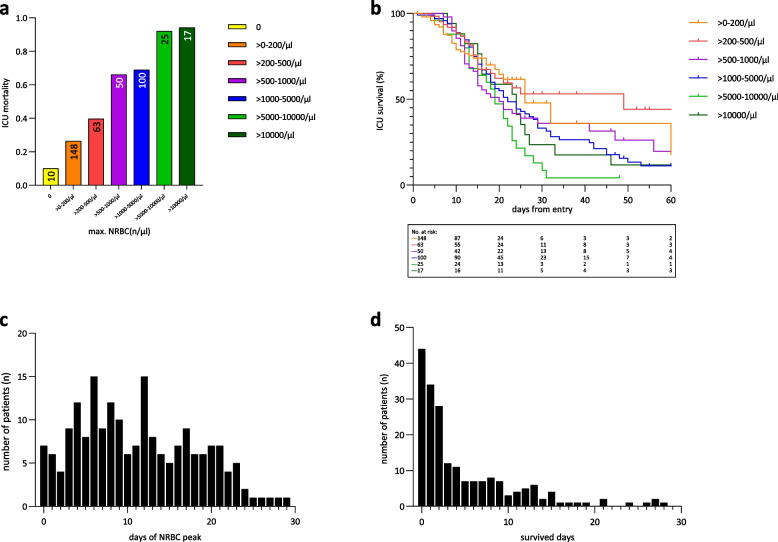
**a **ICU mortality rates in different NRBC groups. ICU mortality of patients with SARS-CoV-2-induced ARDS in relation to the maximum concentration of nucleated red blood cells (NRBCs) in the peripheral blood. Number of patients in the respectively NRBC group are presented in the bar chart, *n* = 413. **b **Survival grouped by different NRBC values. Probability of survival depicted as Kaplan–Meier curves of SARS-CoV-2-induced ARDS patients grouped by different NRBC levels. **c **Days of individual NRBC peak in deceased patients during first 30 days on ICU. Data are presented as histogram indicating the number of patients in relation to the day of the NRBC peak. **d **Survived days after NRBC peak in deceased patients. Number of patients and their survived days after reaching the individual NRBC peak value. Data are presented as histogram indicating the number of patients in relation to survived days

Mortality rates increased with rising NRBC levels, reaching 94% in patients with NRBC peak values above 10,000/µl, whereas those with no measurable NRBC count had a mortality rate of only 10% (Fig. 3a). Grouping patients by increasing maximum NRBC levels also revealed a corresponding increase in mortality rates over time, as indicated by the respective Kaplan–Meier estimates (Fig. 3b). To gain further insight, the timing of individual NRBC peaks and mortality was analyzed. Most deceased patients experienced their peak NRBC levels within the first 10 days of ICU admission (Fig. 3c). Furthermore, among deceased patients, the majority passed away within 24 h after reaching their individual NRBC peak value (Fig. 3d), with a median survival time of 2 days (0; 8) after the NRBC peak.

###  Predictive validity and NRBC cutoff values

**Fig. 4:**
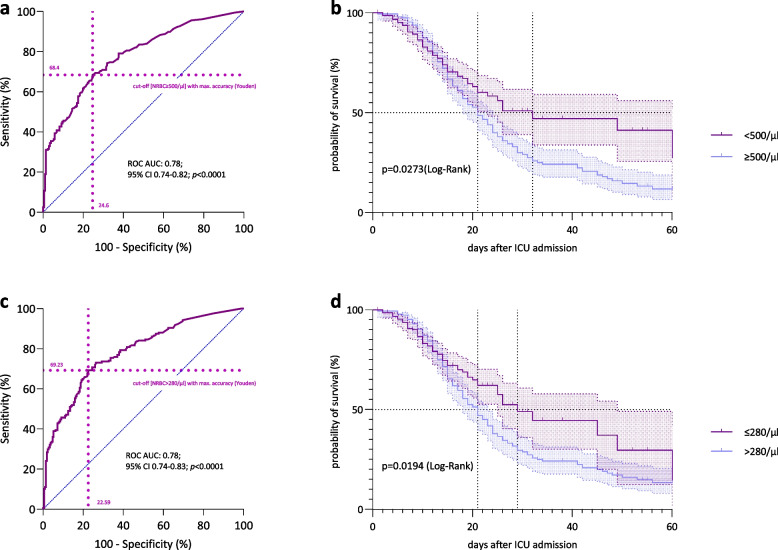
**a **Cut-off value by NRBC maximum. Receiver operator curve (ROC) for the determination of predictive validity of NRBC measurements. The highest sum of sensitivity and specifity was used for calculating a cut-off value. ROC AUC: 0.78; 95% CI 0.74–0.82; *p* < 0.0001. **b **Survival grouped by NRBC cut-off level ≥ 500/µl. Probability of survival depicted as a Kaplan–Meier curve of patients with SARS-CoV-2-induced ARDS grouped by NRBC cut-off level of ≥ 500/µl from ROC analysis. Log Rank test, *p < 0.05, n = 413, median survival < 500/µl: 32 days, ≥ 500/µl: 21 days. **c **Cut-off value by ∆NRBC per 24 h. Receiver operator curve (ROC) for the determination of predictive validity of NRBC measurement increase in 24 h. ROC AUC: 0.78; 95% CI 0.74–0.83; *p* < 0.0001. **d **Survival grouped by NRBC cut-off level > 280/µl (ΔNRBC per 24 h). Probability of survival depicted as a Kaplan–Meier curve of patients with SARS-CoV-2-induced ARDS grouped by NRBC cut-off level increase of > 280/µl in 24 h from ROC analysis. Log Rank test, *p < 0.05, n = 413, median survival ≤ 280/µl: 29 days, > 280/µl: 21 days

Using Youden’s method, a cut-off value for NRBC of ≥ 500/µl was determined to best stratify risk, effectively dividing the study population into two groups with the most significant difference in ICU mortality (ROC area under the curve (AUC) 0.78; 95% CI 0.74–0.82; *p* < 0.0001) (Fig. 4a). The maximum NRBC levels differed significantly between the two groups (maximum NRBC < 500/µl: median 100/µl [20; 4500] vs. maximum NRBC ≥ 500/µl: median 1870 [960; 4500], *p* < 0.0001). Logistic regression analysis with mortality as the dependent variable showed NRBC values of ≥ 500/µl to be an independent risk factor for mortality, with an almost fivefold increased risk of ICU death (odds ratio (OR) 4.72; [95% CI 2.95 – 7.62]; *p* < 0.0001) (Table 2). The probability of survival is illustrated through Kaplan–Meier curves, indicating that patients with NRBC levels below the threshold of 500/µl had a significant survival advantage (median survival 32 days [95% confidence interval (CI) 8.7–43.3] vs. 21 days [95% CI 18.2–23.8], log-rank test, *p* = 0.0273) (Fig. 4b). Reaching the respective cut-off value on the day of ICU admission is already significant, albeit with only modest predictive power (AUC 0.56; 95% CI 0.51–0.62; *p* = 0.0343). However, the predictive value of the cut-off value becomes more substantial for the first 10 days after ICU admission (AUC 0.69; 95% CI 0.64–0.74; *p* < 0.0001), reaching its maximum validity when considering the entire ICU stay (AUC 0.78; 95% CI 0.74–0.82; *p* < 0.0001).

**Table 2:** Multivariate logistic/COX regression analyses of risk factors influencing ICU mortality (cut-off NRBC ≥ 500/µl)

**Multivariate logistic regression**	***p*-value**	**OR**	**95% CI**
ECMO	0.0085	2.13	1.21–3.74
Charlson Comorbidity Index	< 0.0001	1.29	1.14–1.46
CRP	0.0003	1.004	1.002–1.007
NRBC ≥ 500/µl	< 0.0001	4.33	2.55–7.45
**COX regression**			
Charlson Comorbidity Index	0.0008	1.13	1.05–1.20
Septic shock at admission	0.0022	1.64	1.19–2.25
NRBC ≥ 500/µl	0.0076	1.59	1.14–2.25

Parameters considered in the multivariate regression models were ECMO (yes/no); procalcitonin; maximum CRP; maximum IL-6; maximum leukocytes; Charlson Comorbidity Index; septic shock at admission (yes/no), NRBC (yes/no ≥ 500/µl). Data of 413 patients were included. NRBC cutoff ≥ 500/µl was used for analysis

*OR* odds rati, *CI* confidence interval, *HR* hazard ratio, *NRBC* nucleated red blood cells, *ECMO* extracorporeal membrane oxygenation, *CRP* C-reactive protein, *IL-6* Interleukin-6

We investigated the potential correlation between rapidly increasing NRBC levels over a defined timeframe and outcomes. Employing Youden’s method, we determined a threshold for ∆NRBC of > 280/µl per 24 h as a predictor for mortality (ROC AUC: 0.78; 95% CI 0.74–0.83; *p* < 0.0001) (Fig. 4c). In addition, an increase of NRBC value of > 280/µl in 24 h was identified as an independent risk factor for mortality, showing a more than fivefold increased risk of ICU death (OR 5.51; [95% CI 3.45 – 8.90]; *p* < 0.0001) (Table 3). Kaplan–Meier survival curves further supported these findings, revealing a median survival of 29 days in patients with ∆NRBC values ≤ 280/µl, compared to patients above the threshold of 280/µl with a median survival time of 21 days (log-rank test, *p* = 0.0194) (Fig. 4d). Results of multivariate logistic regression and COX regression analyses assessing risk factors for ICU mortality are presented in Table 2 and 3, respectively. An additional subgroup analysis of septic patients showed similar results. Reaching the NRBC cut-off value of 500/µl and an increase of > 280/µl per 24 h was identified as an independent risk factor for ICU mortality in this subgroup of septic patients (supplementary file).

**Table 3:** Multivariate logistic/COX regression analyses of risk factors influencing ICU mortality (cut-off ∆NRBC > 280/µl/24 h)

**Multivariate logistic regression**	***p*-value**	**OR**	**95% CI**
ECMO	0.0039	2.27	1.30–3.98
Charlson Comorbidity Index	0.0001	1.28	1.13–1.46
CRP	0.0015	1.004	1.001–1.006
NRBC > 280/µl	< 0.0001	5.16	3.03–8.92
**COX regression**			
Charlson Comorbidity Index	0.0006	1.13	1.05–1.21
Septic shock at admission	0.0015	1.67	1.21–2.28
NRBC > 280/µl	0.0051	1.62	1.16–2.28

Parameters considered in the multivariate regression models were ECMO (yes/no); procalcitonin; maximum CRP; maximum IL-6; maximum leukocytes; Charlson Comorbidity Index; septic shock at admission (yes/no), NRBC (yes/no > 280/µl). Data of 413 patients were included. NRBC cut-off > 280/µl was used for analysis

*OR* odds ratio, *CI* confidence interval, *HR* hazard ratio, *NRBC* nucleated red blood cells, *ECMO* extracorporeal membrane oxygenation, *CRP* C-reactive protein, *IL-6* Interleukin-6

### Clinical parameters between groups and over the course of time

**Fig. 5:**
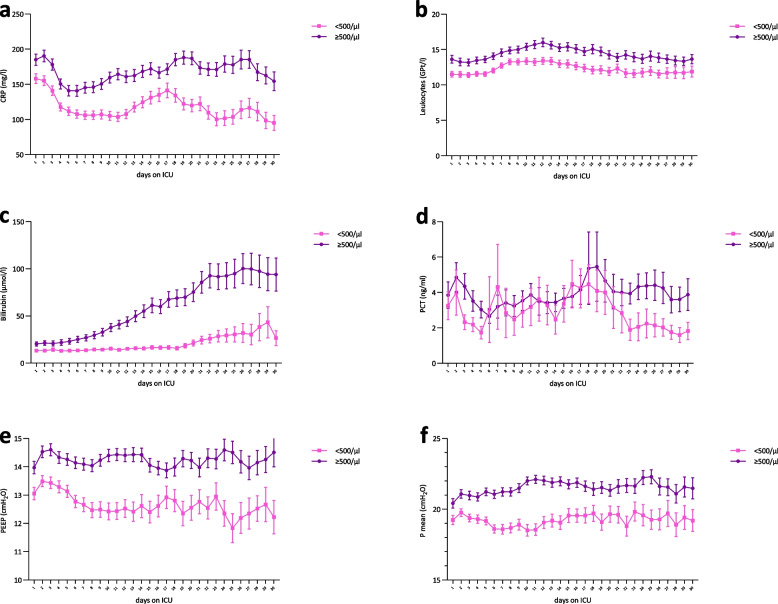
Clinical parameters between groups and over the course of time.** a** 30 days trend mean CRP (mg/l) grouped by NRBC cut-off value ≥ 500/µl, n = 413. **b** 30 days trend mean leukocytes (GPt/l) grouped by NRBC cut-off value ≥ 500/µl, n = 410. **c** 30 days trend mean Bilirubin (µmol/l) grouped by NRBC cut-off value ≥ 500/µl**,** n = 408. **d** 30 days trend mean PCT (ng/ml) grouped by NRBC cut-off value ≥ 500/µl, n = 411. **e** 30 days trend mean PEEP (cmH2O) grouped by NRBC cut-off value ≥ 500/µl, n = 412. **f** 30 days trend Pmean (cmH2O) grouped by NRBC cut-off value ≥ 500/µl, n = 412. Data are presented as mean ± standard deviation (SD)

Clinical parameters showed significant differences between patients with NRBC values < 500/µl and those with ≥ 500/µl. Patients with NRBC ≥ 500/µl exhibited longer ICU stays, prolonged mechanical ventilation durations, and a higher incidence of ECMO usage with extended ECMO durations. While baseline characteristics and pre-existing conditions did not differ significantly, laboratory parameters such as CRP, procalcitonin, leukocytes, and bilirubin were notably elevated in the ≥ 500/µl group. Importantly, over time, these parameters consistently remained higher in patients with NRBC values above the threshold of 500/µl, indicating a sustained impact on clinical outcomes. Moreover, ventilatory variables, including Pmean and PEEP, showed significant differences between the two groups over time, with consistently higher values observed in patients with NRBC values ≥ 500/µl, suggesting a sustained impact on respiratory support needs (Table 1; Fig. 5 a-f).

**Fig.6:**
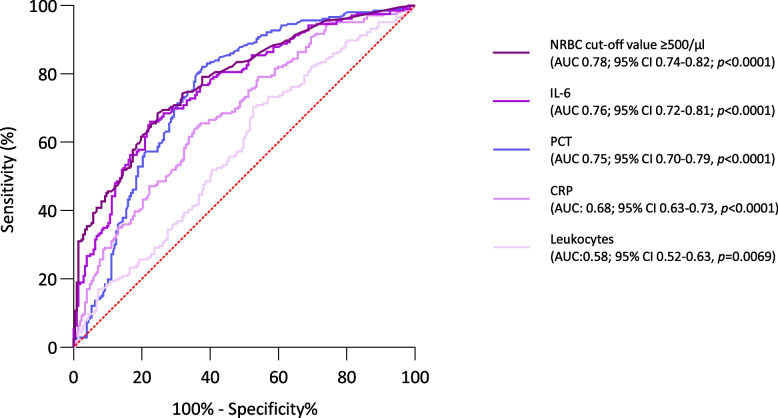
Predictive validity of inflammatory parameters comparison. Receiver operator curves (ROC) for the determination of predictive validity of inflammatory parameters compared to predictive validity of NRBC cut-off-value ≥ 500/µl

We also used ROC analysis for these inflammatory parameters such as IL-6, PCT, CRP and leukocytes to evaluate their diagnostic performance compared to the results of the ROC analysis of the NRBC cut-off value ≥ 500/µl. All parameters seem to be a possible marker for prediction in patients with SARS-CoV-2-induced ARDS, thus the AUC differs remarkably (IL-6 ROC AUC 0.76; 95% CI 0.72–0.81, *p* < 0.0001 vs. PCT ROC AUC 0.75; 95% CI 0.70–0.79; *p* < 0.0001 vs. CRP ROC AUC 0.68; 95% CI 0.63–0.73; *p* < 0.0001 vs. leukocytes ROC AUC 0.58; 95% CI 0.52–0.63, *p* = 0.0069) (Fig. 6).

### NRBC values and longterm outcome

**Fig. 7:**
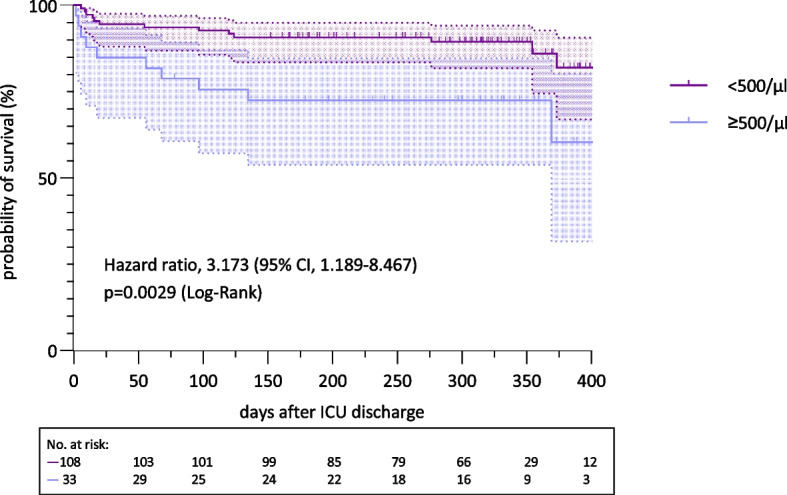
Longterm survival grouped by NRBC cut-off value ≥ 500/µl. Probability of One Year survival depicted as a Kaplan–Meier curve of patients with SARS-CoV-2-induced ARDS grouped by NRBC cut-off level increase of ≥ 500/µl from ROC analysis. Log-rank test, *p* = 0.0029, n = 141, median survival < 500/µl: undefined, > 500/µl: 372 days; HR 3.17 (95% CI 1.19–8.47)

Long-term data were available for 141 of the 413 patients in the study, with 24 deaths recorded. Among these 24 deceased patients, 11 belonged to the group with NRBC values ≥ 500/µl. Kaplan–Meier survival analysis revealed a significant survival disadvantage for patients who had reached the NRBC threshold of ≥ 500/µl during their ICU stay, with a more than threefold increased risk of mortality, ever long after discharge from ICU (median survival 489 days, log-rank test, *p* = 0.0029, hazard ratio (HR) 3.2, 95% CI 1.2–8.5) (Fig. 7). Patients’ characteristics of this subgroup are presented in Table 4.

**Table 4:** Patient characteristics of longterm-survivors

	all patients (n = 141)	NRBC < 500/µl (*n* = 108)	NRBC ≥ 500/µl (*n *= 33)	*p*-value
*Demographics*				
Age (years)	60 (50; 67.5)	62 (53; 69.8)	55 (45; 62.5)	0.001
Male sex, *n* (%)	91 (64.5)	72 (66.7)	19 (57.6)	0.0006
Body mass index (kg/m2)	31 (27; 36)	31 (27; 34.8)	34 (26; 39.5)	n.s
Septic shock at admission, *n* (%)	23 (16.3)	17 (15.7)	6 (18.2)	n.s
ARDS severity, *n* (%)				n.s.*
Mild	1 (0.7)	1 (0.93)	0 (0)	
Moderate	24 (17)	20 (18.52)	4 (12.1)	
Severe	116 (82.3)	87 (80.6)	29 (87.9)	
*ICU characteristics*				
Spontaneous breathing at admission, *n* (%)	38 (26.9)	32 (29.6)	6 (18.2)	n.s
Intubated at admission, *n* (%)	103 (73.1)	76 (70.4)	27 (81.8)	n.s
Tracheotomy at admission, *n* (%)	9 (6.4)	6 (5.6)	3 (9.1)	n.s
Mechanical ventilation (days)	12 (7; 21)	10 (6; 15.8)	25 (15; 44)	< 0.0001
ECMO, *n* (%)	43 (30.5)	22 (20.4)	21 (63.6)	< 0.0001
ECMO duration (hours)	342 (165; 633)	211.3 (126.3; 422.9)	598.5 (286; 1026)	0.0018
ICU length of stay (days)	14 (9; 23)	13 (8; 18)	26 (16.5; 46.5)	< 0.0001
RRT,* n* (%)	19 (13.5)	11 (10.2)	8 (24.2)	0.0119
RRT duration (hours)	237 (101; 359)	263.5 (110.5; 429.6)	166.3 (84.4; 264.7)	n.s
ICU mortality, *n* (%)	24 (17)	13 (12.0)	11 (33.3)	0.0076
*Ventilation parameters*				
pH	7.39 (7.34; 7.44)	7.4 (7.34; 7.44)	7.38 (7.33; 7.44)	n.s
PaCO_2_ (kPa)	6.4 (5.8; 7.15)	6.4 (5.8; 6.8)	7.1 (5.5; 8.2)	n.s
P/F ratio	67.5 (52.5; 90)	75 (54.4; 90)	60 (48.8; 78.8)	0.0371
Pmean (cm H_2_O)	20 (18; 23)	20 (18; 23)	21 (18; 23)	n.s
PEEP (cm H_2_O)	14 (12; 15)	14 (12; 15)	14 (12; 15)	n.s
SpO_2_ (%)	94 (91; 96)	94 (91; 97)	93 (91; 96)	n.s
*Laboratory parameters*				
D-dimers max (ng/ml)	4000 (3076; 4000)	3939 (2624; 4000)	4000 (3925; 4000)	n.s
Bilirubin µmol/l	20 (11.9; 41.5)	17.25 (11.1; 33.6)	50.6 (17.9; 104.2)	< 0.0001
CRP (mg/l)	243 (154.7; 327.1)	224.9 (144.5; 315.7)	290.8 (218.5; 357.5)	0.0053
PCT (ng/ml)	1.2 (0.49; 3.8)	0.9 (0.4; 2.3)	2.4 (1.2; 8.3)	0.0017
IL-6 maximum value, (pg/ml)	302 (116; 635)	269.5 (94.9; 518.3)	468 (165.5; 1480)	0.006
Leukocytes maximum value (GPt/L)	19.65 (14.5; 27.4)	17.9 (13.2; 23.7)	27.6 (23.3; 31.1)	< 0.0001
*Excerpt from pre-existing conditions*				
Arterial hypertension, *n* (%)	97 (68.8)	77 (71.3)	20 (60.6)	n.s
Cardiovascular diseases, *n* (%)	66 (46.8)	49 (45.4)	17 (51.5)	n.s
Neurovascular/Stroke/ICH, *n* (%)	10 (7.1)	8 (7.4)	2 (6.1)	n.s
CHD, *n* (%)	16 (11.4)	14 (12.9)	2 (6.1)	n.s
COPD, *n* (%)	9 (6.4)	7 (6.5)	2 (6.1)	n.s
Other chronic lung diseases, *n* (%)	16 (11.4)	14 (12.9)	2 (6.1)	n.s
Nicotine abuse, *n* (%)	13 (9.2)	11 (10.2)	2 (6.1)	n.s
Diabetes, *n* (%)	56 (39.7)	42 (38.9)	14 (42.4)	n.s
Chronic kidney disease, *n* (%)	15 (10.6)	11 (10.2)	4 (12.1)	n.s
Chronic dialysis, *n* (%)	1 (0.7)	1 (0.9)	0 (0)	n.s
Charlson Comorbidity Index (CCI)	2 (1;4)	2 (1; 4)	2 (1; 4)	n.s

Discrete variables are presented as absolute numbers, median or percentage and were analyzed with Fisher’s exact test. Continuous variables are presented as median and (25; 75) percentiles and were analyzed with the Mann–Whitney U test for independent groups

n.s. = not significant

*NRBC* nucleated red blood cells; *ARDS* acute respiratory distress syndrome; *ICU* intensive care unit; *ECMO* extracorporeal membrane oxygenation; *RRT* renal replacement therapy; *PaCO*_*2*_ arterial partial pressure of carbon dioxide*; PaO*_*2*_ arterial partial pressure of oxygen; *SpO*_*2*_ peripheral oxygen saturation; *CRP* C-reactive protein; *PCT* Procalcitonin; *IL-6* Interleukin-6; *ICH* intracranial hemorrhage; *COPD* chronic obstructive pulmonary disease, *CHD* coronary heart disease

^*^significance for ARDS severity moderate vs. severe, mild was excluded due to *n* = 1

## Discussion

In patients with SARS-CoV-2-induced ARDS, the presence of NRBC in peripheral blood is significantly associated with increased ICU mortality and predicts outcomes with high prognostic power. Our study identified a cut-off level of ≥ 500/µl NRBC to effectively stratify risk, with values above this threshold linked to an almost fivefold risk of ICU death. Furthermore, our study shows that a solitary increase in NRBC levels of above 280/µl over a 24-h period is associated with a high mortality rate of 75% and a more than fivefold risk for ICU death.

These findings are consistent with previous studies regarding the predictive value of NRBC in critically ill patients with ARDS [13, 15, 23–25]. In contrast to other studies, the prevalence of NRBCs in our cohort was notably higher at 97.6%, regardless of whether they were associated with COVID-19 or ARDS [14, 15, 23–25]. This increased prevalence may be attributed to the impact of SARS-CoV-2 infection on bone marrow function, leading to leukoerythroblastic reactions [26, 27]. However, the exact mechanisms leading to the appearance of NRBCs in the peripheral blood, particularly in the context of COVID-19, remain unclear. Potential triggers such as systemic inflammation and hypoxemia may release NRBCs into the peripheral blood [14, 23, 28]. These triggers can also be found in critically ill patients with ARDS [29]. We previously hypothesized that these factors might affect and amplify NRBC levels in ARDS patients due to similar pathomechanisms [24]. Our recent findings within a COVID-19-ARDS cohort lend support to this hypothesis.

In our study, the determined threshold of ≥ 500/µl NRBC is notably higher than those reported in other studies [24, 25]. Schmidt et al. investigated the predictive value of NRBCs in a smaller cohort of 206 patients with SARS-CoV-2-induced ARDS and identified a threshold of 105/µl NRBC to best stratify risk, which is lower than the threshold reported herein [25]. In their cohort, which may differ in terms of clinical parameters and severity of pre-existing conditions, the authors consider the maximum NRBC count to be a late predictor of ICU mortality due to the fact that established clinical scoring systems such as Sequential Organ Failure Assessment score (SOFA), Acute Physiology And Chronic Health Evaluation (APACHE II), P/F ratio, and IL-6 and PCT values occurred significantly earlier than the individual NRBC peak in their study group. However, in our findings, most of the deceased patients reached their individual peak in the first 5–10 days of ICU stay, which is relatively early. The fact that most of the deceased patients died within 24 h after their individual peak is not only consistent with the results of other studies in similar cohorts, but also emphasizes the potential importance of elevated NRBC levels as an early predictor of mortality.

In our results, inflammatory parameters, especially Il-6 and PCT, also seem to be possible predictive markers, although the receiver operating curve characteristics of NRBC cut-off value ≥ 500/µl are significantly higher regarding this study group. A combined analysis of inflammatory markers and NRBC values in relation to mortality may be a possible approach for early prediction not only in patients with SARS-CoV-2-induced ARDS. Further studies are warranted to provide a deeper understanding of whether NRBCs serve as an early predictor in critical care settings.

In our study, we identified an absolute threshold as a robust predictor of mortality, with significantly higher mortality rates observed upon reaching this threshold at any time during the ICU stay. In addition, we implemented a ∆NRBC assessment per day based on individual changes in NRBC counts over a 24-h period. Our findings indicate an enhanced predictive value of ∆NRBC compared to absolute NRBC values. We speculate that the absolute change in NRBC counts over time, i.e., the dynamic development of these cell counts in the peripheral blood, might be more informative than absolute numbers.

Despite its recognized prognostic value in critically ill patients during the ICU stay, NRBC positivity upon ICU admission did not exhibit significant predictive power for mortality in our study. Our findings suggest that its prognostic value may manifest later in the disease course rather than at the onset of critical illness. In our cohort, the NRBC threshold was typically reached around 5 to 10 days after ICU admission, which may be attributed to the evolving course of the disease or the progression of inflammation. Interestingly, this time frame roughly aligns with an observed increase in CRP and leukocyte values between days 5 and 10 in the NRBC group above the threshold in our study. In a subgroup analysis in patients with septic shock, our findings demonstrate that increasing levels of NRBC remain an independent risk factor for patients with SARS-CoV-2-induced ARDS, regardless of their septic shock status. However, due to the limited size of our dataset and its retrospective character, any interpretation regarding the relationship between NRBC levels and disease progression during the early phase of ICU admission is speculative. Nevertheless, we have shown that regardless of when the patient’s NRBC value reaches the cut-off value of ≥ 500/µl during the ICU stay, it is significantly associated with a worse outcome. This also holds true for the day of ICU admission. However, despite the challenges in interpreting NRBC levels upon ICU admission, integrating NRBC assessment into clinical practice might facilitate early identification of patients at risk. Further research is needed to validate the clinical utility of NRBCs and their effectiveness in guiding therapeutic strategies.

To our knowledge, our study is the first to investigate the predictive value of NRBC for long-term outcomes in patients with SARS-CoV-2-induced ARDS. We found that NRBC levels ≥ 500/µl were associated with an increased risk of death even one year after ICU discharge. These findings are in line with Purtle and colleagues, who previously showed that NRBCs predict post-discharge mortality and unplanned hospital readmission 90 days after ICU discharge, and advocate for a more intensive follow-up program for at-risk patients [30]. Comparing these studies with ours highlights the consistent trend for NRBCs to serve as a prognostic marker for mortality in critically ill patients, regardless of the underlying cause of their condition.

Our study has limitations. First, the retrospective nature of our analysis may introduce inherent biases and limitations associated with data collection and analysis. Second, the relatively small sample size of our cohort may have restricted the statistical power and generalizability of our findings. Furthermore, the single-center nature of our study may limit the extrapolation of our results to broader patient populations or different settings. The absence of data regarding the disease course before admission, including NRBC measurement, and variations in the timing of admission to our hospital may have influenced the results. In addition, ARDS and COVID-19 patients were specifically transferred to the University Hospital Dresden as a tertiary referral center for differentiated lung support and ECMO therapy, which may lead to a selection bias.

Larger-scale studies with diverse patient populations and prospective designs may be worthwhile to validate our findings and provide further insight into the role of NRBC as a prognostic marker in critically ill patients.

## Conclusion

NRBCs predict mortality in patients with SARS-CoV-2-induced ARDS with high prognostic power. Integrating NRBC assessment into prognostic models for patients with SARS-CoV-2-induced ARDS may hold promise for improving risk stratification and guiding therapeutic interventions.

### Supplementary Information

Supplementary Material 1.

## Data Availability

The data sets analyzed in the current study are available from the corresponding author on reasonable request.

## Abbreviations

APACHE II: Acute Physiology And Chronic Health Evaluation
ARDS: acute respiratory distress syndrome
AUC: area under the curve
CCI: Charlson Comorbidity Index
CI: confidence interval
CR: C-reactive protein
ECM: extracorporeal membrane oxygenation
HR: hazard ratio
ICH: intracranial hemorrhage
ICU: intensive care unit
NRB: nucleated red blood cells
OR: odds ratio
PCT: procalcitonin
PEEP: positive end-expiratory pressure
Pmean: mean airway pressure
RKI: Robert Koch Institute
ROC: receiver operating curves
RRT: renal replacement therapy
SARS-CoV-2: severe acute respiratory syndrome coronavirus type 2
