# Supplementary material for: Nucleated red blood cells as a prognostic marker for mortality in patients with SARS-CoV-2-induced ARDS: an observational study
Source: J Anesth Analg Crit Care. 2024 Jun 28;4:38. doi: 10.1186/s44158-024-00174-2 (PMC11212412; doi:10.1186/s44158-024-00174-2)
Supplement: Supplementary file 1 — Supplementary Material 1. [file 44158_2024_174_MOESM1_ESM.docx]

**Supplementary file** Multivariate logistic analyses of risk factors influencing ICU mortality in a sub-group with patients with septic shock (cut-off NRBC ≥500/µl)

| **Multivariate logistic regression** | *p*-value | OR | 95% CI |
| --- | --- | --- | --- |
| ECMO | 0.041 | 0.18 | 0.03-0.89 |
| Charlson Comorbidity Index | 0.012 | 1.65 | 1.15-2.55 |
| NRBC ≥ 500/µl | 0.039 | 4.26 | 1.11-17.99 |

Parameters considered in the multivariate regression models were ECMO (yes/no); procalcitonin; maximum CRP; maximum IL-6; maximum leukocytes; Charlson Comorbidity Index, NRBC (yes/no ≥500/µl). Data of 92 patients were included. NRBC cutoff ≥500/µl was used for analysis. *OR* odds ratio; *CI* confidence interval; *HR* hazard ratio; *NRBC* nucleated red blood cells; *ECMO* extracorporeal membrane oxygenation; *CRP* C-reactive protein; *IL-6* Interleukin-6

Dear reviewer,

Thank your for your …

1. Regarding your first statement, we fully agree that a multivariate ROC and COX analysis is necessary to better clarify whether the increasing NRBC levels during SARS-CoV-2 infection is an independent prognostic factor for clinical outcome or just a concomitant phenomenon with other elevated inflammatory markers. Therefore, we performed a multivariate logistic regression and COX analysis including the following parameters: ECMO, PCT, CRP, IL-6, leukocytes, CCI, septic shock at admission, NRBC cut-off value and updated Table 2 in the manuscript.

We show that reaching the NRBC cutoff-value of 500/µl (and 280/µl) is still an independent risk factor for mortality during ICU stay and is not influenced by other inflammatory markers.

2. Thank you…..

In a subgroup analysis using a multivariate logistic regression model in patients with septic shock we found out that….adding in to the manuscript in a sentence???

6. We changed from “COVID-19-induced” to “SARS-CoV-2 induced” in the manuscript.

Reviewer 2:
